# Clinical and Genetic Analysis of *SMARCC2*‐Related Diseases in Three Chinese Patients

**DOI:** 10.1002/mgg3.70198

**Published:** 2026-01-14

**Authors:** Shan Ou, Shujie Zhang, Qi Yang, Qiang Zhang, Xunzhao Zhou, Qinle Zhang, Xiuliang Rong, Nana Qi, Jiale Qian, Bibing Xi, Ranran Lin, Shengkai Wei, Jingyu Su, Zailong Qin, Jingsi Luo

**Affiliations:** ^1^ Guangxi Key Laboratory of Birth Defects Research and Prevention, Guangxi Key Laboratory of Reproductive Health and Birth Defects Prevention Maternal and Child Health Hospital of Guangxi Zhuang Autonomous Region Nanning China; ^2^ Department of Genetic and Metabolic Central Laboratory Maternal and Child Health Hospital of Guangxi Zhuang Autonomous Region Nanning China; ^3^ Guangxi Clinical Research Center for Pediatric Diseases Maternal and Child Health Hospital of Guangxi Zhuang Autonomous Region Nanning China; ^4^ Department of Neonatology Maternal and Child Health Hospital of Guangxi Zhuang Autonomous Region Nanning China; ^5^ Department of Child Health Care Maternal and Child Health Hospital of Guangxi Zhuang Autonomous Region Nanning China

**Keywords:** intellectual disability and developmental delay, *SMARCC2* variant, SMARCC2‐related disease, whole exome sequencing

## Abstract

**Background:**

Coffin‐Siris syndrome (CSS) is a rare, clinically and genetically heterogeneous disorder characterized by coarse facial features, microcephaly, intellectual disability (ID), developmental delay (DD), and hypo/aplastic digital nails and phalanges, typically of the 5th digit. CSS is an autosomal dominant disease resulting from mutations in genes encoding components of BRG1/BRM‐associated factor (BAF) chromatin remodeling complexes. More than 300 CSS patients have been reported with variants in genes in the BAF pathway. Recently, patients carrying *SMARCC2* variants have been reported to be associated with CSS8. However, as the number of cases increases, many patients do not exhibit the representative clinical symptoms of CSS. Additional case reports and clinical studies will contribute to a redefinition of SMARCC2‐related disorders.

**Methods:**

In this research, three patients with SMARCC2‐related disorders from China were recruited. Genomic DNA was extracted from the peripheral blood leukocytes of these patients' parents and other family members, and then subjected to whole‐exome sequencing as well as Sanger sequencing.

**Results:**

In the present study, two de novo variants (c.1311‐3C>G, c.347G>A (p.Arg116His)) and a novel de novo variant (c.346C>T (p.Arg116Cys)) in the *SMARCC2* gene were detected in three patients with neurodevelopmental disorders by whole exome sequencing. The clinical presentation of our patients supports a redefinition of SMARCC2‐related diseases, which include mild to moderate DD, mild ID, facial dysmorphism, mild speech delay, hypotonia, feeding difficulties, brain abnormalities, attention deficit hyperactivity disorder (ADHD), and autistic behaviors. Furthermore, both the type of variant and its specific location may be contributing factors influencing the clinical outcomes.

**Conclusion:**

Our study expands the genetic spectrum of *SMARCC2* variants and detailed genotypic and phenotypic descriptions are important for the diagnosis of SMARCC2‐related disease and accurate clinical management.

## Graphical abstract

In this article, we investigate the pathogenesis of three unrelated Chinese children who exhibited mild to moderate developmental delay, mild intellectual disability, facial dysmorphism, mild speech delay, hypotonia, feeding difficulties, brain abnormalities, attention deficit hyperactivity disorder (ADHD), and autistic behaviors. Furthermore, both the type of variant and its specific location may be contributing factors influencing the clinical outcomes. Our study expands the genetic spectrum of SMARCC2 variants, and detailed genotypic and phenotypic descriptions are important for the diagnosis of SMARCC2‐related disease and accurate clinical management.

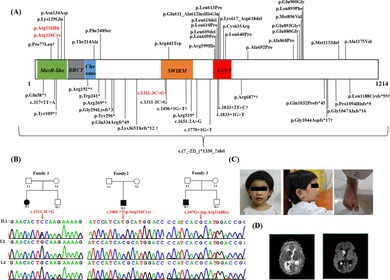

**Abbreviations**
CSS: Coffin‐Siris syndrome
CSS‐8: Coffin‐Siris syndrome 8
DD: Developmental delay
ID: Intellectual disability
NMD: Nonsense‐mediated mRNA decay
SNV: Single nucleotide variants
WES: Whole exome sequencing

## 1 Introduction

Coffin‐Siris syndrome (CSS) is a rare group of neurodevelopmental disorders (NDD) with an estimated incidence of 1:10,000–1:100,000. It is characterized by a range of developmental delay (DD), intellectual disability (ID), growth retardation, microcephaly, speech impairment, coarse facial features, hypertrichosis, sparse scalp hair, hypoplastic nails of the fifth finger and/or toe, and brain anomalies, the most prominent of which is hypoplasia or agenesis of the corpus callosum (Kosho et al. 2013; Coffin and Siris 1970). According to available reports, the clinical and genetic manifestations of individuals with CSS are heterogeneous, with most cases being sporadic and showing autosomal dominant inheritance (Kosho et al. 2014). CSS is caused by variants in genes encoding components of the ATP‐dependent chromatin remodeling BRG1‐associated factor (BAF) complex (the mammalian SWI/SNF complex). Within this family of genes, de novo pathogenic variants in *ARID1A*, *ARID1B*, *ARID2*, *DPF2*, *SMARCC2*, *SMARCA4*, *SMARCE1*, and *SMARCB1* have been identified in patients with CSS (Santen, Aten, et al. 2012; Tsurusaki et al. 2012; Vasileiou et al. 2018; Wieczorek et al. 2013; Van Houdt et al. 2012; Bramswig et al. 2017; Machol et al. 2019). In addition, de novo variants in the *SOX11* and *SOX4* genes were observed in individuals with clinical phenotypes consistent with CSS (Tsurusaki et al. 2014; Zawerton et al. 2019). Several of these genes are also associated with other ID/NDD (Santen, Kriek, and van Attikum 2012). Thus, additional reports on variants in these genes will help us better understand the phenotype spectrum and the relationship between genotype and phenotype.

**FIGURE 1:**
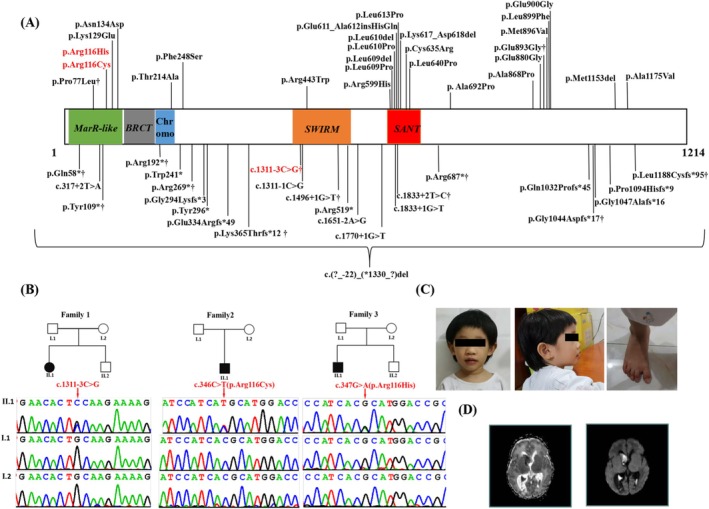
Clinical and genetic features. (A) Mutational landscape of SMARCC2. Boxes = functional domains. All 48 reported variants are shown. The variants in our patients are highlighted in red. ^†^Shared by more than one patient. (B) Pedigrees and Sanger sequencing of families 1, 2 and 3 with pathogenic variants. (C) Photograph of patient 1 (family 1, II‐1) at the age of 3 years and 4 months old showing macrocephaly and abnormality of the right pinna. (D) Axial slices of T1‐weighted images (T1WI) and T2WI acquired at 14 days in patient 2 (family 2, II‐1) show hydrocephalus, dilation of the right lateral ventricle and subependymal cerebral hemorrhage.

Recently, heterozygous variants in the SWI/SNF‐related, matrix‐associated, actin‐dependent regulator of chromatin subfamily c member 2 (*SMARCC2*) gene were described as underlying Coffin‐Siris syndrome 8 (CCS‐8; MIM: 618362), characterized by ID and multiple malformations (Machol et al. 2019). The *SMARCC2* gene, located at 12q13.3, encodes a core subunit of the chromatin remodeling complex BAF, BAF170. The protein SMARCC2 consists of MarR‐like (10–136), BRCT (140–183), SWIRM (424–521 aa) and SANT domains (596–647 aa) and plays an essential role in embryogenesis and cardiac development (Hota et al. 2019). To date, more than 60 individuals with *SMARCC2* gene variants have been identified (Figure 1A, Table S1; Machol et al. 2019; Li et al. 2022, 2025; Yi et al. 2022; Carss et al. 2014; Sun et al. 2022; Chen et al. 2022; Lo et al. 2022; Gofin et al. 2022; Bosch et al. 2023). The phenotypes of these patients are heterogeneous, including mild to severe ID, growth retardation, prominent speech delay, behavioral abnormalities, hypotonia, feeding difficulties, skin problems, brain abnormalities, and dysmorphic features, including hypertrichosis, thick eyebrows, thin upper and thick lower vermillion, and upturned/anteverted nostrils. These features overlap with those observed in other NDD, such as CSS and Nicolaides‐Baraitser syndrome. Thus, additional reports on these gene variants will help us better understand the spectrum of the resulting phenotypes and the genotype–phenotype relationship. Herein, we report two de *novo* variants (c.1311‐3C>G, c.347G>A (p.Arg116His)) and a novel de *novo* variant (c.346C>T (p.Arg116Cys)) in the *SMARCC2* gene (NM_003075.3) in three Chinese patients with NDD (Figure 1B), and further provide a detailed description of the associated clinical features of these patients.

## 2 Materials and Methods

### 2.1 Ethical Compliance

The present study conformed with the tenets of the Declaration of Helsinki and was approved by the Institutional Review Board and Ethics Committee of Guangxi Maternal and Child Health Hospital. Written informed consent was obtained from the patients' families.

### 2.2 Whole‐Exome Sequencing and Sanger Sequencing

Genomic DNA extraction was performed using a Lab‐Aid DNA kit sourced from Zeesan Biotech Co. Ltd. (Xiamen, China). The DNA concentrations were subsequently measured with a NanoDrop 1000 spectrophotometer (Thermo Fisher Scientific Inc.). In each proband, whole‐exome sequencing (WES) was performed to detect any potential pathogenic mutations. Whole‐exome capture was performed using the Agilent SureSelect Human All Exon v.6 kit (Agilent Technologies, Santa Clara, CA), and sequencing was performed with a HiSeq 2500 system (Illumina, San Diego, CA, USA) with a read depth > 100× and more than 95% of the targeted regions covered over 20×. The obtained sequenced reads were aligned to the human genome assembly GRCh37 by employing the Burrows‐Wheeler Aligner software. Subsequently, variants were annotated through the application of Genome Analysis Toolkit 3.4 (GATK, www.broadinstitute.org/gatk) and TGex software (LifeMap Sciences, Alameda, VA, USA). Variants with minor allele frequencies > 1% in public (e.g., 1000 Genomes Project, Exome Sequencing Project, gnomAD and ExAC) as well as in‐house databases were excluded. Following that, synonymous and intronic variants that did not impact splicing sites were also excluded. Finally, four pathogenicity prediction tools, including SIFT, PolyPhen‐2, CADD, and MutationTaster, were used to predict the potential pathogenicity of the identified variants. A 3D model of the SMARCC2 protein was constructed using SWISS‐MODEL (https://swissmodel.expasy.org/). The pathogenicity of the identified variants in each patient was assessed according to the American College of Medical Genetics and Genomics and the Association for Molecular Pathology (ACMG/AMP) guidelines (Richards et al. 2015).

## 3 Results

### 3.1 Clinical Features

Patient 1, a 3‐year‐4‐month‐old female, was the first child of healthy, non‐consanguineous Chinese parents (Figure 1A, Table 1). She was born at 38^+4^ weeks of gestation with normal weight and length. Her development was delayed as she started sitting at 13 months and walking at 25 months. She started talking at 2 years old, and she currently speaks a few words of Chinese, can communicate in short sentences, and understands simple commands in Chinese. She was 3 years and 4 months old at the most recent examination, at which she had postnatal growth retardation with macrocephaly (> 3 SD). She had dysmorphic facial features with hypertelorism, a wide nasal bridge, and an abnormality of the right pinna. Dysmorphic features also included small hands and feet and a sandal gap between the first and second toe (Figure 1C). Her Full‐Scale IQ was 75, as assessed by the Chinese Wechsler Intelligence Scale for Children. Her brain MRI, X‐ray of the chest and spine, echocardiography, and abdominal ultrasound examinations were normal, and she experienced no seizures. Her G‐band karyotype was determined to be 46, XX.

**TABLE 1:** Clinical features of the patient with de novo *SMARCC2* mutations.

Clinical features	Patient 1	Patient 2	Patient 3
Variants in *SMARCC2* (NM_003075.3)	c.1311‐3C>G	c.346C>T (p.Arg116Cys)	c.346C>T (p.Arg116His)
Inheritance	De novo	De novo	De novo
Gender	Female	Male	Male
Age at last examination	3 years and 4 months	21 days	9 years and 8 months
Gestation	Full‐term	36^+3^ weeks	Full‐term
Short stature	< −1.5 SD	NO	< −1.5SD
Macrocephaly	> 3 SD	NO	NO
Developmental delay/movement delay	Yes; mild	Yes; mild	Yes; mild
Intellectual disability	Yes; mild	Yes; mild	Yes; mild
Hypotonia	Yes	Yes	Yes
Age of walking	25 months	NA	18 months, gait ataxia
Age of first words	24 months	NA	28 months
Behavior anomalies	NO	NA	Attention Deficit Hyperactivity Disorder, autistic behaviors, irritability and frequent crying, frequent hand biting, repetitive speech and movements
Facial dysmorphisms	Hypertelorism, wide nasal bridge, and abnormality of the right pinna	NO	NO
Other anomalies	Small hands and feet and a sandal gap between the first and second toe	Hydrocephalus, dilation of the right lateral ventricle, and subependymal cerebral hemorrhage	

Patient 2, the third child born to healthy, non‐consanguineous parents from Guangxi, China, was a 21‐day‐old male neonate referred to the Guangxi maternal and Child Health Center (Guangxi, China) for genetic counseling due to jaundice and hydrocephalus (Figure 1B, Table 1). He was born at 36^+3^ weeks gestation with normal measurements (length: 50 cm, > 90th; weight: 3080 g, 50th–75th; head circumference: 34 cm, 50th). He presented with hypotonia, feeding difficulties, a weak cry, and a poor suck. At 2 weeks of age, he was noted to have poor head control and poor responsiveness. A brain MRI at the age of 14 days showed hydrocephalus, dilation of the right lateral ventricle, and subependymal cerebral hemorrhage (Figure 1D). His G‐band karyotype was determined to be 46, XY.

Patient 3 was first seen at the Department of Child Health Care, Guangxi Zhuang Autonomous Region Maternal and Child Health Hospital at the age of 9 years and 8 months due to learning difficulties (Figure 1C, Table 1). He was the first‐born male child of healthy, non‐consanguineous parents, born at 39 weeks and 3 days of gestation with normal birth weight and length. His developmental milestones were mildly delayed. He began to walk independently at 18 months but continues to exhibit gait ataxia. He also had hypotonia. He initiated expressive language at 2 years and 4 months, and is capable of basic verbal communication, although with unclear articulation and an inability to formulate complex sentences. The most recent physical examination revealed that his height is below the normal standard (< −1.5SD). According to the assessment using the Chinese version of the Wechsler Intelligence Scale for Children, his full‐scale intelligence quotient (FSIQ) is 69. He was diagnosed with Attention Deficit Hyperactivity Disorder (ADHD) and autistic behaviors. He was emotionally unstable, with characteristics of irritability and frequent crying. He had significant impairment in social interaction as evidenced by frequent hand biting, repetitive speech and movements with behavioral problems and hyperactivity.

### 3.2 Molecular Analysis

**FIGURE 2:**
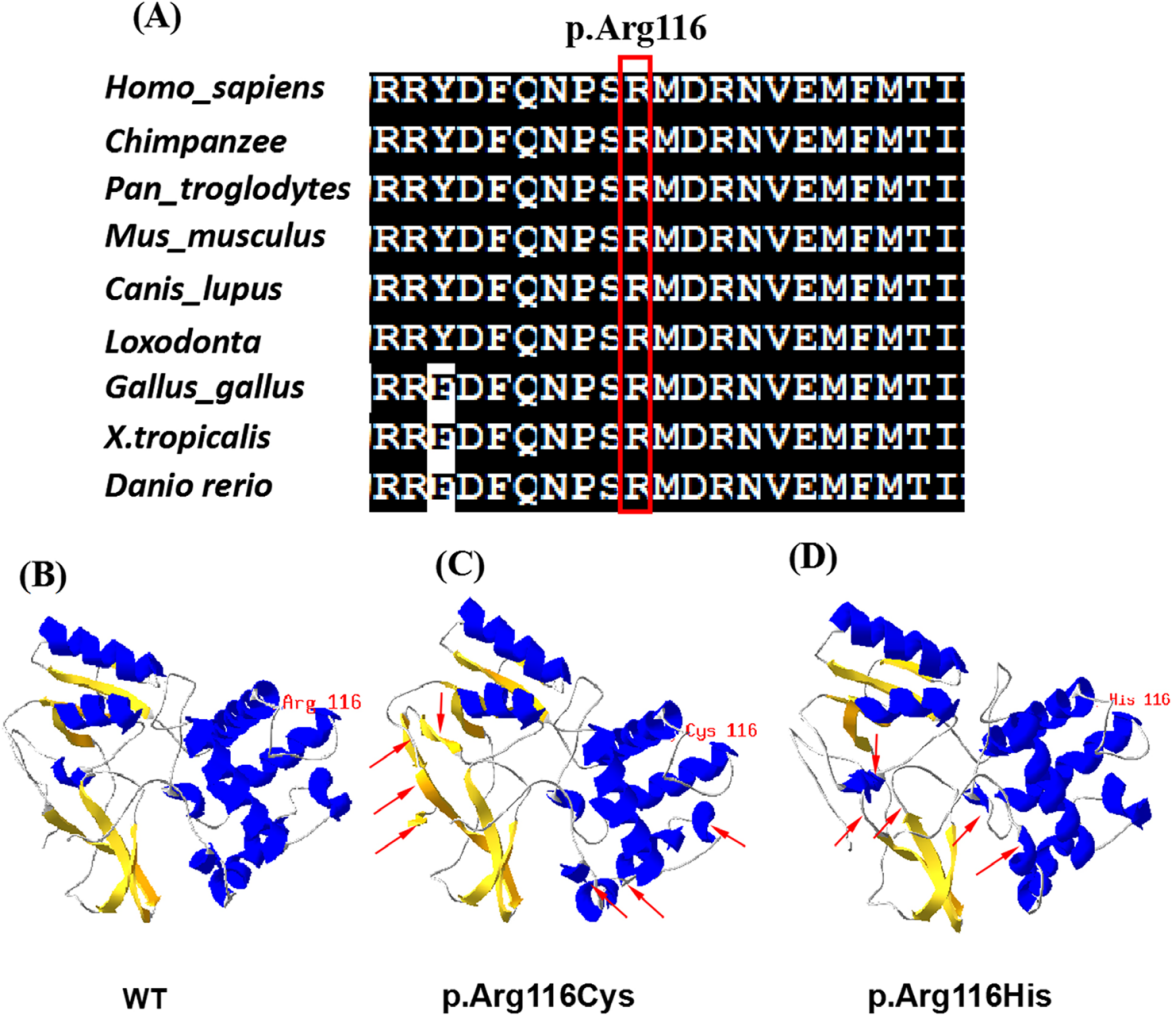
(A) Multispecies alignment showing the strong conservation of SMARCC2 p.Arg116. (B–D) Three‐dimensional structures of SMARCC2 protein. (B) Wild type; (C) c.346C>T (p.Arg116Cys) mutant‐type, three‐dimensional structure modeling predicted an increase in the proportion of β‐sheets regions, and a decrease in α‐helix and random coil regions in mutant protein; (D) c.347G>A (p.Arg116His) mutant‐type, three‐dimensional structure modeling predicted an increase in the proportion of α‐helix and random coil regions, and a decrease in β‐sheet regions in mutant protein. The dimer alterations are indicated by an arrow.

Using WES, we detected heterozygous variants in the *SMARCC2* gene (NM_003075.3) in the probands as follows: c.1311‐3C>G in patient 1, c.346C>T (p.Arg116Cys) in patient 2 and c.347G>A (p.Arg116His) in patient 3 (Figure 1B). We validated the three variants by Sanger sequencing and sequenced the parental samples to identify the variants as de novo. The variant c.347G>A (p.Arg116His) is present in ClinVar (RCV003443429.1). It has been observed in genome samples where it failed the random forest filters, and it has not been detected in exome samples. Specifically, c.346C>T (p.Arg116Cys) is a novel variant, which was not deposited in the Human Gene Mutation database, gnomAD, 1000 Genomes Project, Exome Sequencing Project, ExAC, ClinVar, and the Single Nucleotide Polymorphism databases. The variant c.346C>T (p.Arg116Cys) and c.347G>A (p.Arg116His) were located in the fourth exon of the *SMARCC2* gene and the MarR‐like domain of the SMARCC2 protein. Multiple sequence alignment revealed that residue 116 is well conserved (Figure 2A). The variant c.346C>T (p.Arg116Cys) and c.347G>A (p.Arg116His) were predicted to be deleterious by multiple *in silico* methods, including SIFT, PolyPhen‐2, CADD, and MutationTaster. The three‐dimensional structures of wild type (WT) and mutant SMARCC2 proteins were predicted using SWISS‐MODEL. The results indicated that, compared to the WT protein, the proportion of regions folded into β‐sheets increased, while the proportions of α‐helices and random coils decreased in the p.Arg116Cys mutant protein. Domains 13–15, 23–25, 41–42, 82, and 255–256 were predicted to be altered from α‐helices into random coils, 18 and 31–33 were predicted to be altered from random coils into α‐helices; 189, 258–260, 264–266, and 272–274 were predicted to be altered from random coils into β‐sheets; 257 was predicted to be altered from α‐helices into β‐sheets (Figure 2C). In the p.Arg116His mutant protein, the proportions of regions folded into α‐helices and random coils increased, and the proportion of β‐sheets decreased. Domains 12, 187–188 and 255–256 were predicted to be altered from random coils into α‐helices, 190–191 and 205–206 were predicted to be altered from β‐sheets into random coils (Figure 2D). However, the c.1311‐3C>G variant was previously reported in an individual with CCS‐8. This mutation leads to abnormal RNA splicing and reduced RNA expression levels, causing loss of function. Table 2 presents the pathogenicity analysis and classification of the three SMARCC2 variants according to ACMG/AMP criteria.

**TABLE 2:** Predicted pathogenicity of de novo *SMARCC2* variants.

Patient	Variant (NM_003075.3)	Inheritance	Mutationtaster	PolyPhen‐2	SIFT	CADD	Splice‐AI	ACMG/AMP
Patient 1	c.1311‐3C>G	DNV	N.A.	N.A.	N.A.	N.A.	0.99	P (PVS1 + PS2 + PM2 + PP3)
Patient 2	c.346C>T (p.Arg116Cys)	DNV	D	D	D	28.4	N.A.	LP (PS2 + PM2 + PP3)
Patient 3	c.346C>T (p.Arg116His)	DNV	D	D	D	32.4	N.A.	LP (PS2 + PM2 + PP3)

Abbreviations: D, deleterious or damaging; DNV, de novo variant; N.A., not available; P, pathogenic; PD, probably damaging.

## 4 Discussion

*SMARCC2*‐related disease is a very rare NDD caused by variants in the *SMARCC2* gene. In 2014, Carss et al. reported a *SMARCC2* variant in a female fetus with abdominal situs inversus, cardiac malposition of the great arteries, and multiple ventricular septal defects (Carss et al. 2014). Machol et al. further expanded the disease phenotype by studying *SMARCC2* variants in 15 patients with neurodevelopmental (NDD) abnormalities and multiple malformations (Machol et al. 2019). To date, more than 60 patients with SMARCC2‐related disease have been reported (Table S1; Machol et al. 2019; Li et al. 2022, 2025; Yi et al. 2022; Carss et al. 2014; Sun et al. 2022; Chen et al. 2022; Lo et al. 2022; Gofin et al. 2022; Bosch et al. 2023). The clinical presentation of these subjects with SMARCC2‐related disease includes varying degrees of DD/ID, speech impairment, growth retardation, behavioral abnormalities, hypotonia, brain abnormalities, feeding difficulties, and dysmorphic features. In the current study, we performed WES and identified two de novo variants in the *SMARCC2* gene in three unrelated Chinese patients. These patients exhibit developmental abnormalities associated with NDD in multiple domains similar to those seen in SMARCC2‐related disorders, including mild to moderate DD, mild ID, facial dysmorphism, mild speech delay, hypotonia, feeding difficulties, brain abnormalities, attention deficit hyperactivity disorder (ADHD), and autistic behaviors. In addition, to our knowledge, hydrocephalus was reported for the first time (Table 1).

The splice‐site variant of c.1311‐3C>G has been previously reported in an individual with CCS‐8. Machol et al. demonstrated that the splice‐site mutation c.1311‐3C>G resulted in the absence of protein production with a significant decrease in mRNA level due to nonsense‐mediated degradation (Machol et al. 2019). Both the c.346C>T (p.Arg116Cys) and c.347G>A (p.Arg116His) variants were de novo missense variants. Multiple *in silico* tools predicted deleterious outcomes of these missense variants. Three‐dimensional structural analysis of the SMARCC2‐R116C and SMARCC2‐R116H proteins revealed that the mutation alters the secondary and tertiary structures by changing the number of local α‐helices and β‐sheets in the protein, which may affect structural stability (Figure 2B–D). According to the ACMG/AMP standards and guidelines (Li et al. 2025), the c.1311‐3C>G variant is pathogenic according to the PVS1, PS2, PM2_supporting, and PP3 criteria (Table 2). In contrast, both the c.346C>T (p.Arg116Cys) and c.347G>A (p.Arg116His) variants are classified as likely pathogenic on the basis of PS2, PM2, and PP3 criteria (Table 2).

To date, a total of 48 *SMARCC2* variants (including our variants) associated with NDD have been reported, including 29 missense/in‐frame variants and 29 truncating variants (e.g., nonsense/frameshift variants and splicing variants that cause protein frameshifts) (Table S1). Notably, patients carrying the missense/in‐frame variant appear to be more likely to have more severe and extensive clinical manifestations (Bosch et al. 2023). The SMARCC2 protein comprises five domains, namely the MarR‐like domain (10–136), the BRCT domain (140–183), the Chromo domain (189–217), the SWIRM domain (424–521) that mediates specific protein–protein interactions, and the SANT domain (596–647) involved in chromatin binding of the protein (UniProt protkb/Q8TAQ2). It is noteworthy that the majority of the reported missense/in‐frame variants are specifically located within the SANT domain, and all variants identified in this domain are missense or in‐frame. Among the four variants recorded in the SWIRM domain, three were also missense/in‐frame variants. Patients carrying missense/in‐frame variants in the SANT and SWIRM domains tend to exhibit more severe and extensive clinical manifestations compared to those with missense/in‐frame variants in other domains. These results suggest that the severity of disease caused by *SMARCC2* variants is not only related to the type of variant but also to the specific domain in which the variant is located. Moreover, this also indicates that *SMARCC2* variants may not cause disease solely through haploinsufficiency. For missense/in‐frame variants, there may be other pathogenic mechanisms, such as dominant‐negative effects. In addition, patients carrying the same variant may also exhibit diverse phenotypes. For instance, patients 1 and 7 both harbored the same splice‐site variant c.1311‐3C>G in the SWIRM domain. Patient 7 exhibited significant DD, characterized by severe DD, prominent speech impairment, behavioral abnormalities, kyphosis, and epilepsy. In contrast, Patient 1 in this study presented with a milder phenotype. These results are currently limited by the number of reported cases and identified variants. As the patient cohort expands, it is anticipated that the phenotypic spectrum will be further refined, and additional insights into genotypic effects and other phenotypic determinants will emerge. Further functional studies of these variants are essential to enhance our understanding of the disease and its mechanisms of action.

Recently, Bosch et al. conducted a summary analysis of the clinical features of 65 patients with SMARCC2‐related diseases and found that these patients mainly presented with common clinical features such as varying degrees of intellectual disability/developmental delay (ID/DD), speech delay, behavioral abnormalities, hypotonia, feeding difficulties, brain abnormalities, and nonspecific dysmorphic facial features (Gofin et al. 2022). Notably, representative manifestations of CSS, such as hypertrichosis, sparse scalp hair, nail hypoplasia, and characteristic fifth finger/toe bone hypoplasia or absence, are rarely observed in these patients. Based on these significant differences in clinical phenotypes, the researchers propose that classifying SMARCC2‐related diseases as a subtype of CSS may not accurately reflect their unique pathological characteristics. In our patient cohort, only Patient 1 exhibited hypertelorism, a broad nasal bridge, and right auricular abnormalities, while Patients 2 and 3 showed no significant facial dysmorphism. Additionally, none of the hallmark features of CSS were observed in our cases. These findings support the necessity of classifying SMARCC2‐associated disorders as a distinct entity separate from CSS.

It is unclear how these variants cause ID/DD, speech delay, behavioral abnormalities, hypotonia, feeding difficulties, coarse facial appearance, brain abnormalities, and additional clinical symptoms. Multiple mechanisms have been hypothesized to explain SMARCC2‐related DD/ID. The SMARCC2 protein interacts with BAF47 (SMARCB1), BAF57 (SMARCE1), BAF155 (SMARCC1)/BAF170 (SMARCC2), and BAF60 A/B/C (SMARCD1/2/3) to form the core BAF complex (mammalian SWI/SNF) (Phelan et al. 1999). The SWI/SNF complex plays an essential role in chromatin and transcriptional regulation, which affects many biological processes (Wilson and Roberts 2011). *SMARCC2* is a chromatin remodeling gene involved in autism spectrum disorder (Ben‐David and Shifman 2013). In addition, SMARCC2 plays a key role in embryonic development and cortical development. It determines the size of the mammalian body and cortex (Tuoc et al. 2013). Furthermore, *SMARCC2* variants affect the expression of genes including *H19*, *SCRG1*, *RELN*, and *CACNB4*, which are involved in regulating neuronal development and function (Machol et al. 2019). Further functional studies of these variants are needed to determine disease mechanisms.

## 5 Conclusion

In conclusion, three de novo variants in the SMARCC2 gene were detected in three Chinese patients affected by SMARCC2‐related diseases. This study expands the mutation spectrum of CSS‐8 syndrome. This is the first report of the c.346C>T (p.Arg116Cys) mutation in the *SMARCC2* gene. Detailed clinical features and molecular diagnoses will further aid our understanding of the genotype–phenotype correlation of SMARCC2 pathogenic variants and SMARCC2‐related diseases. The heterogeneous phenotypes observed in these patients support the need for a redefinition of SMARCC2‐related diseases. Additionally, the type of variant and its specific location may also influence clinical outcomes. More case reports and further functional studies of these variants are crucial for deepening our understanding of the disease and its underlying mechanisms.

## Author Contributions

Shan Ou and Jingsi Luo designed the study. Shan Ou and Jingsi Luo gathered clinical information from the family members and drafted the manuscript. Shujie Zhang, Qi Yang, Qiang Zhang, Qinle Zhang, Xunzhao Zhou, Xiuliang Rong, Nana Qi, Jiale Qian, Bibing Xi, Ranran Lin, Shengkai Wei, and Jingyu Su performed the sequencing, as well as analyzed and interpreted the data. Shan Ou, Zailong Qin, and Jingsi Luo revised the manuscript. All authors coordinated the study coordination and revised the manuscript. All authors read and approved the final version of the manuscript.

## Funding

This research was supported by the Guangxi Natural Science Foundation under Grant (2024GXNSFBA010072), the Health Department of Guangxi Province (Grant No. Z‐A20220256), the Guangxi Key Laboratory of reproductive health and birth defect prevention (21‐220‐22), Guangxi Clinical Research Center for Pediatric Diseases (Guike ad22035121), the Young Scientists Fund of the National Natural Science Foundation of China (No. 82201312); the Health Department of Guangxi Zhuang Autonomous Region (Z‐A20230362, Z20210440, and Z‐A20240323).

## Ethics Statement

All procedures in this study were approved by the Institutional Review Board and Ethics Committee of Guangxi Maternal and Child Health Hospital, and were conducted according to the medical ethics defined in the Declaration of Helsinki. Written informed consent for the publication of data was obtained from the patients' parents.

## Consent

Written informed consents for publication of clinical details and clinical images were obtained from all the participants and for those younger than 16 years old, obtained from their parents.

## Conflicts of Interest

The authors declare no conflicts of interest.

## Supporting information


**Table S1:** Summary of the clinical features of the patients.

## Data Availability

The data that support the findings of this study are openly available in NCBI at https://www.ncbi.nlm.nih.gov/sra/PRJNA902508.
